# Plan quality and delivery time comparisons between volumetric modulated arc therapy and intensity modulated radiation therapy for scalp angiosarcoma: A planning study

**DOI:** 10.1002/jmrs.239

**Published:** 2017-07-29

**Authors:** Yudai Kai, Ryo Toya, Tetsuo Saito, Akiko Kuraoka, Yoshinobu Shimohigashi, Yuji Nakaguchi, Masato Maruyama, Ryuji Murakami, Yasuyuki Yamashita, Natsuo Oya

**Affiliations:** ^1^ Department of Radiological Technology Kumamoto University Hospital Kumamoto Japan; ^2^ Department of Radiation Oncology Kumamoto University Hospital Kumamoto Japan; ^3^ Department of Human Oncology University of Wisconsin School of Medicine and Public Health Madison Wisconsin USA; ^4^ Department of Medical Imaging Faculty of Life Sciences Kumamoto University Kumamoto Japan; ^5^ Department of Diagnostic Radiology Kumamoto University Hospital Kumamoto Japan

**Keywords:** Angiosarcoma, intensity modulated radiation therapy, scalp irradiation, volumetric modulated arc therapy, X‐ray Voxel Monte Carlo algorithm

## Abstract

**Introduction:**

Due to its spherical surface, scalp angiosarcoma requires careful consideration for radiation therapy planning and dose delivery. Herein, we investigated whether volumetric modulated arc therapy (VMAT) is superior to intensity modulated radiation therapy (IMRT) in terms of the plan quality and delivery time.

**Methods:**

Three different coplanar treatment plans were created for four patients, comprising a two‐arc VMAT plan as well as 5‐field and 9‐field IMRT plans with 6 MV beams. The X‐ray Voxel Monte Carlo algorithm was employed for dose calculation. A radiation therapy dose of 60 Gy was prescribed to the planning target volume (PTV) in 30 fractions. The homogeneity indexes (HIs) and conformity indexes (CIs) of the PTV, organs at risk (OARs) doses and delivery times were calculated and compared.

**Results:**

For the VMAT, 5‐field and 9‐field IMRT plans, the mean HIs were 0.14, 0.16 and 0.15; CIs_100%_ were 0.63, 0.61 and 0.64; CIs_98%_ were 0.72, 0.66 and 0.70 and CIs_95%_ were 0.74, 0.67 and 0.71 respectively. All mean dose parameters of the VMAT and 9‐field IMRT plans for the brain were equal to or lower than those of the 5‐field IMRT plan. For the 5‐field IMRT plan, the dose constraints for the left lens were not satisfied in two patients. The mean delivery times were 3.3, 11.1 and 14.7 min for the VMAT, 5‐field and 9‐field IMRT plans respectively.

**Conclusion:**

The VMAT plan quality is comparable to that of 9‐field IMRT, with a reduced delivery time. Therefore, VMAT represents a valuable, sophisticated irradiation technique for treating scalp angiosarcoma.

## Introduction

Cutaneous angiosarcoma most frequently arises on the scalp of elderly people.^1^, ^2^ Although surgical resection is the mainstay of treatment, many patients are not candidates for surgery due to old age or coexisting disease. Radiation therapy is commonly performed as an alternative treatment modality in such cases.^1^, ^2^ The goal of irradiation for scalp angiosarcoma is to deliver a high, uniform dose to the target while sparing organs at risk (OARs) such as the brain and optical structures. However, the irregular shape of the target on the spherical scalp surface requires careful consideration for radiation therapy planning and dose delivery.^3^

Traditionally, the lateral photon–electron (LPE) technique was performed for the treatment of scalp angiosarcoma especially if lesions are located over the whole scalp. This technique consists of parallel opposing lateral photon fields which cover the outer rind of the scalp around the top of the head, as well as lateral electron fields which cover the remaining lateral portion of the scalp which are matched with the photon beam.^4^ However, dose heterogeneity due to matching fields which use an electron beam against a target on a spherical scalp was problematic.^3^, ^5^ More recently, brachytherapy has also been introduced, but this technique is not common and is performed only at a limited number of institutions.^3^ Intensity modulated radiation therapy (IMRT), which uses fixed gantry angles and divides each large radiation beam into numerous small beamlets whose intensities are adjusted individually, has been increasingly performed at many institutions.^6^ Wojcicka et al.^3^ compared LPE, IMRT and brachytherapy for the treatment of scalp tumours and concluded that IMRT provided the best target dose homogeneity and conformity with acceptable doses to the OARs.

Another highly sophisticated technique, volumetric modulated arc therapy (VMAT), has also been introduced. Using this method, the gantry is rotated while the dose is being delivered, and three parameters (dose rate, field shape and speed of gantry rotation) can be changed as the beam is rotated.^6^ Some investigators have suggested that VMAT is superior to IMRT for treating tumours of the brain, head and neck, lung, prostate and others.^7^ In terms of the evaluation of VMAT for scalp tumours, Hu et al.^8^ compared LPE, 9‐field IMRT and VMAT (RapidArc; Varian, CA) for the treatment of diffuse sebaceous carcinoma. Their results suggested that VMAT is advantageous in terms of dose homogeneity and conformity of the target as well as the dose to the brain. However, they included the whole skull layer in the clinical target volume (CTV). For the treatment of scalp angiosarcoma, the CTV is usually limited to the thin scalp surface.^9^, ^10^ Furthermore, Hu et al. evaluated plan qualities according to a model‐based algorithm, the anisotropic analytical algorithm (AAA). Radiation therapy to the scalp tumour usually requires a shell for head fixation and a bolus for build‐up of dose at the tumour surface.^3^, ^5^, ^8^ Therefore, unlike other tumour sites, dose calculations for the treatment of scalp tumours must accommodate heterogeneous regions with multi‐layered complex densities of bolus, scalp, skull, cerebral fluid and brain. As the AAA does not take these various densities into account accurately,^11^, ^12^ it may not provide sufficiently precise dose calculations of scalp angiosarcoma, leading to a need for a more accurate dose calculation algorithm.

Recently, a treatment planning system (TPS) based on the X‐ray Voxel Monte Carlo (XVMC) algorithm became commercially available.^13^, ^14^, ^15^ Dose calculation of heterogeneous regions is more accurate with XVMC than with model‐based algorithms.^14^ Additionally, XVMC shows much faster calculation performance compared to general purpose Monte Carlo codes while maintaining calculation accuracy.^15^, ^16^ The purpose of this study was to validate the advantages of VMAT over IMRT for the treatment of scalp angiosarcoma in terms of plan quality, delivery time and monitor units (MUs) by employing the XVMC algorithm in four patients with varying sites and target volumes.

## Materials and Methods

### Patients

This retrospective planning study was approved by the institutional review board of our institution. In 2013, four consecutive patients with scalp angiosarcoma underwent radiation therapy at our institution. The clinical characteristics of the patients are shown in Table 1. All patients gave prior informed consent to treatment and to the use of treatment data in future studies. Images of computed tomography (CT) simulations for radiation therapy planning were used for this study.

**Table 1:** Clinical characteristics of the patients

Patient no.	Sex	Age (years)	UICC stage	Primary lesion location	PTV (cm^3^)
1	Female	61	T2N0M0	Entire scalp	932.8
2	Male	76	T1N0M0	Left parietotemporal	265.7
3	Male	74	T2N0M0	Parietal	215.0
4	Male	81	T2N0M0	Frontoparietal	666.1

UICC, Union for International Cancer Control; PTV, planning target volume.

### CT simulation and target volume definition

Prior to CT simulation, we marked the tumour volume using radio‐opaque wires to allow us to define the gross tumour volume (GTV) on CT images. To obtain an adequate dose to the tumour surface, we used a bolus with a thickness of 1 cm in addition to a head shell for two patients, and an in‐house moulded shell for two patients. We performed simulations using a CT scanner (LightSpeed RT; GE, Amersham, UK) with a slice thickness of 2.5 mm and no gap.

**Figure 1:**
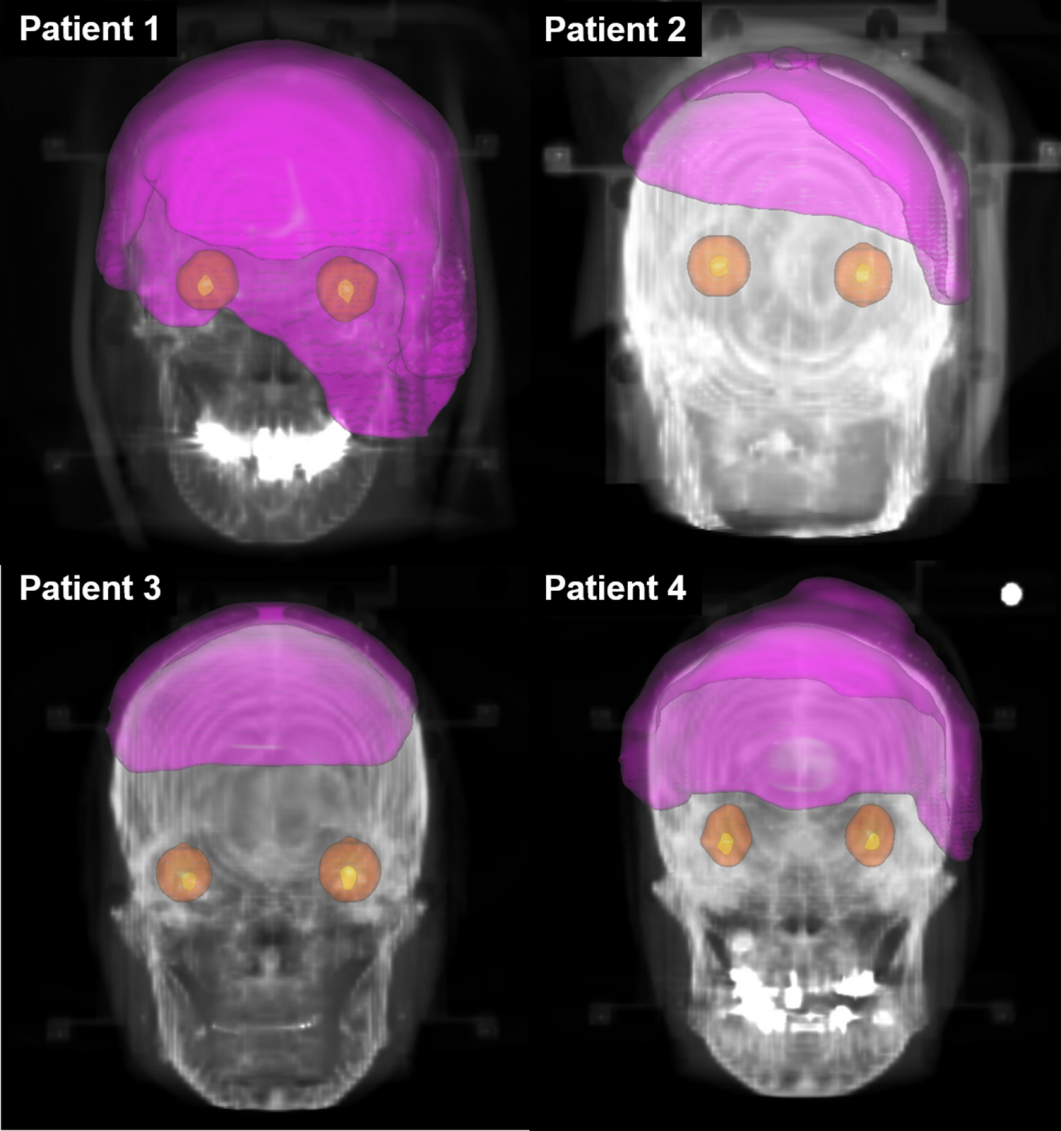
Structures of four patients. The planning target volumes, eyes and lenses are rendered in pink, orange and yellow respectively.

The CTV was defined as the GTV and scalp, plus a 30‐mm margin added to the GTV along the scalp surface.^17^ The planning target volume (PTV) was determined by adding an isotropic 2‐mm margin to the CTV (Fig. 1). The OARs (brain, spinal cord, brain stem, optic chiasm, eyes, lenses and optic nerves) were subsequently contoured.

### Treatment planning

For each patient, three different coplanar treatment plans were created; these comprised a two‐arc VMAT plan and 5‐field and 9‐field step‐and‐shoot IMRT plans with a 6‐MV photon energy. Monaco TPS version 3.20 (Elekta AB, Stockholm, Sweden) was used. XVMC was employed for the dose calculation with an isotropic dose grid size of 3 mm and a standard deviation of 1% per plan as a convergence criterion. Dose calculations were performed as dose to medium. A linear accelerator, Synergy (Elekta AB, Stockholm, Sweden), equipped with an Agility multi‐leaf collimator (MLC) with a leaf width of 5 mm was employed for irradiation. A dose of 60 Gy in 30 fractions was prescribed to the PTV.^3^, ^5^, ^8^ Dose constraints for optimisation are shown in Table 2. The *D*_95%_ values (defined below) of the PTV were maintained at 95% while optimising various other parameters. For each VMAT plan, a collimator angle of 15° was applied. For each 5‐field IMRT plan, gantry angles of 0°, 72°, 144°, 216° and 288° were applied. For each 9‐field IMRT plan, gantry angles of 0°, 40°, 80°, 120°, 160°, 200°, 240°, 280° and 320° were applied. The collimator was not rotated for IMRT plans.^18^ The maximum dose rate for all plans was 600 MU/min.

**Table 2:** Optimisation constraints for VMAT, 5‐field and 9‐field IMRT planning

Structure	Prescription	Constraint
PTV	60 Gy/30 f	*V*_110%_ < 5%
		*D*_95%_ = 95%
		*V*_93%_ > 97%
Brain		*V*_60 Gy_ = 0%
		*V*_45 Gy_ < 20%
		*V*_20 Gy_ < 60%
		*D*_mean_ < 30 Gy
Spinal cord		*D*_max_ < 45 Gy
Brain stem		*D*_max_ < 54 Gy
Optic chiasm		*D*_max_ < 54 Gy
Eyes		*D*_max_ < 45 Gy
Lenses		*D*_max_ < 10 Gy
Optic nerves		*D*_max_ < 54 Gy

VMAT, volumetric modulated arc therapy; IMRT, intensity modulated radiation therapy; PTV, planning target volume; *V*_*x*%_, fractional volume receiving *x*% of the prescribed dose; *D*_*x*%_, absorbed dose received by *x*% of PTV; *V*_*x* Gy_, fractional volume receiving *x* Gy; *D*_mean_, mean dose; *D*_max_, maximum dose.

### Analysis and comparison among plans

Dose parameters of the PTV and OARs were compared in terms of dose constraints for optimisation. Conformity index (CI) and homogeneity index (HI) were used for the evaluation of the PTV. The CI was calculated using the following equation published by Paddick et al.:^19^

$$\mathrm{CI}=\left(V_{\mathrm{Tref}}/V_{T}\right)\times \left(V_{\mathrm{Tref}}/V_{\mathrm{ref}}\right)$$

where *V*_Tref_ is the volume of the target covered by the reference isodose (100%, 98% or 95% of the prescribed dose), *V*_T_ is the target volume and *V*_ref_ is the volume of the reference isodose. The HI was calculated using the following equation published in ICRU Report 83:^20^

$$\mathrm{HI}=\left(D_{2\%}-D_{98\%}\right)/D_{50\%}$$

where *D*_*x*%_ was the absorbed dose received by *x*% of the PTV. For the evaluation of OARs, maximum dose, mean dose and/or *V*_*x* Gy_ were employed, where *V*_*x* Gy_ represents a fractional volume receiving a specific (*x*) dose in Gy. Delivery time and total MUs for each plan were recorded and compared. Delivery time was defined as the time between initiation of the first port and termination of the final port. The order of ports was optimised to minimise the time for gantry rotation between ports.

### Dose verification

Point and relative dose verifications were performed to ascertain that each of the created plans could be accurately delivered. A 0.016 cc micro‐ion chamber (Exradin A14SL; Standard Imaging, Middleton, WI, USA) was connected to an electrometer (RAMTEC 1000 plus; Toyo Medic, Tokyo, Japan) and placed inside a dosimetry phantom (Solid Water; Gammex, Middleton, WI) to measure a point dose at an arbitrarily selected high‐dose region. The criterion for the difference between measured and calculated dose was within 3%. A cylindrical diode array, ArcCHECK (Sun Nuclear, Melbourne, FL), was used to perform relative dose verifications by using the gamma‐index method with a criterion of 3%/3 mm and a threshold dose of 30% of the global maximum dose. The criterion for the gamma pass rate was that it be above 95%.

## Results

**Figure 2:**
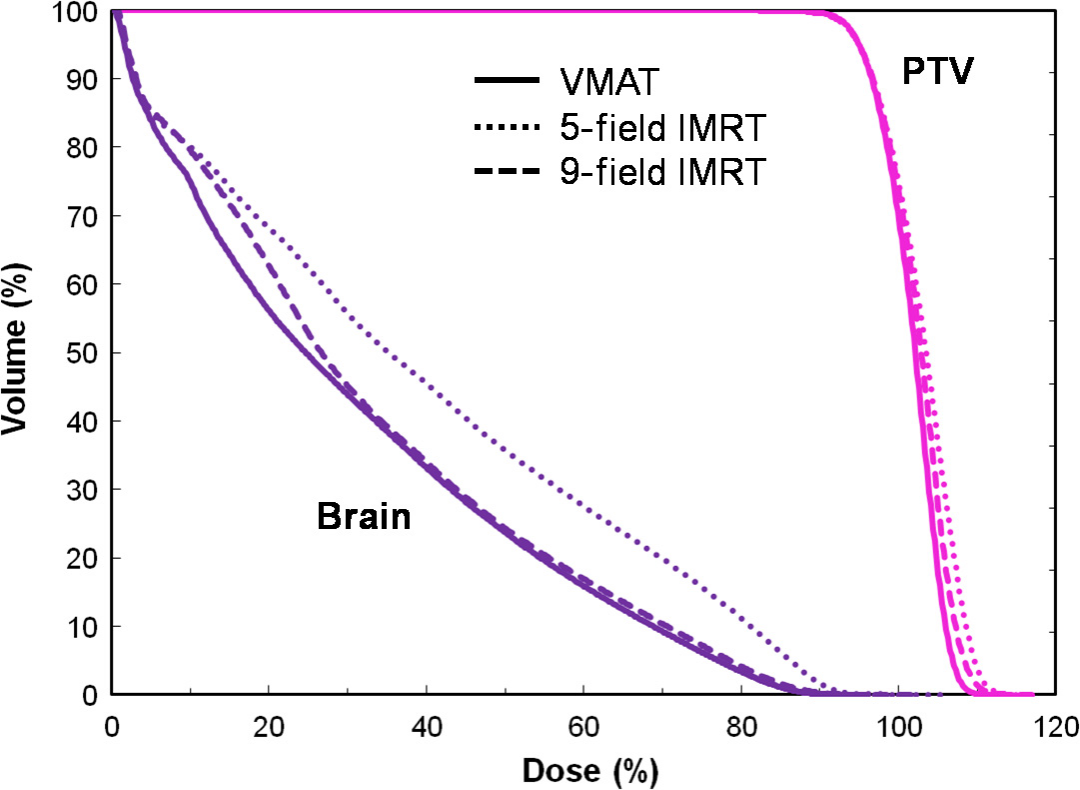
Mean dose–volume histograms of the planning target volumes and brains in four patients. VMAT, volumetric modulated arc therapy; IMRT, intensity modulated radiation therapy; PTV, planning target volume.

**Figure 3:**
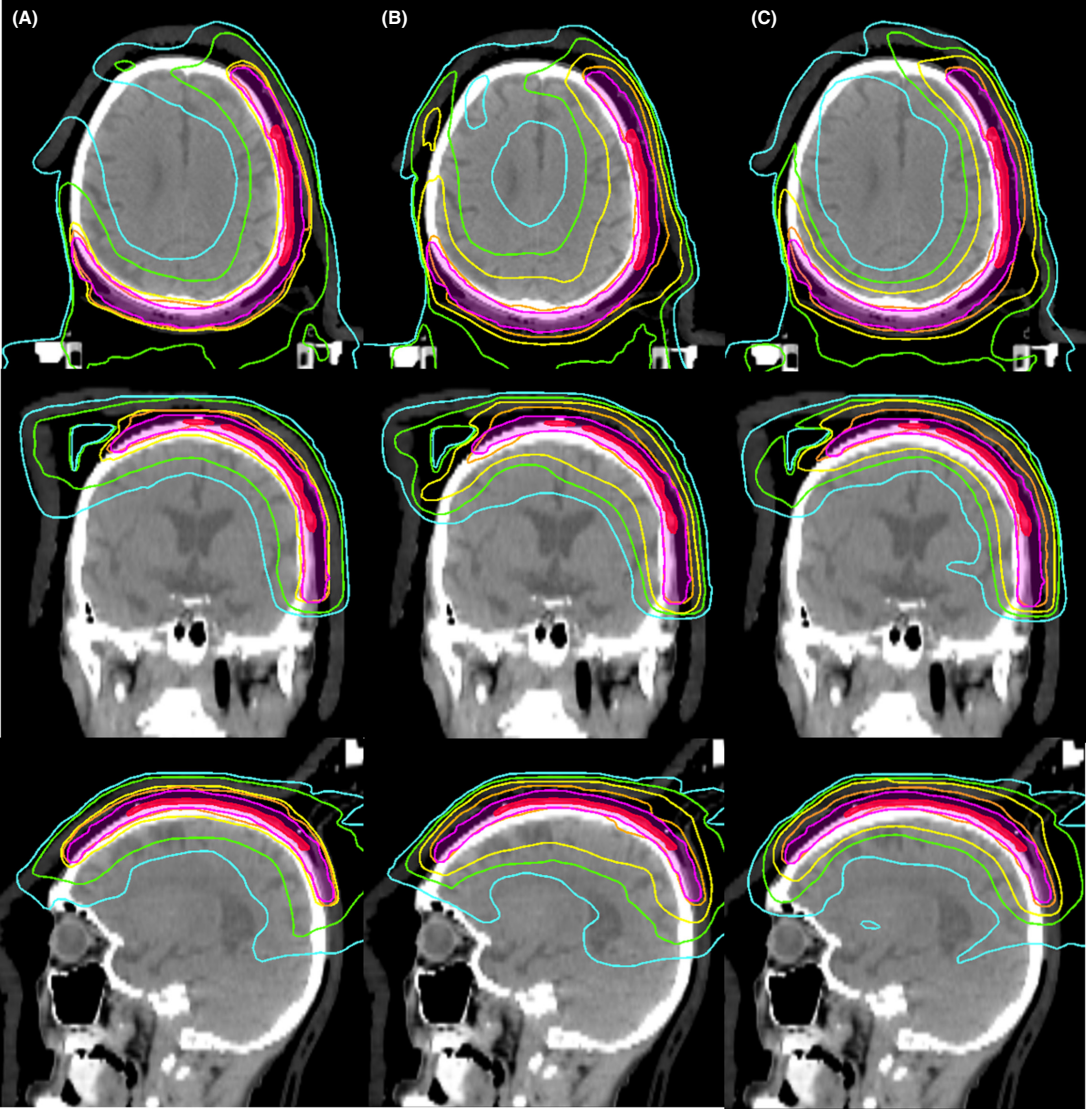
Dose distributions of the three plans in patient 2. (A) Volumetric modulated arc therapy, (B) 5‐field intensity modulated radiation therapy (IMRT) and (C) 9‐field IMRT. The gross tumour volumes and planning target volumes are rendered in red and pink respectively. Isodose lines of 30%, 50%, 70% and 95% of the prescribed dose are rendered in blue, green, yellow and orange respectively.

**Figure 4:**
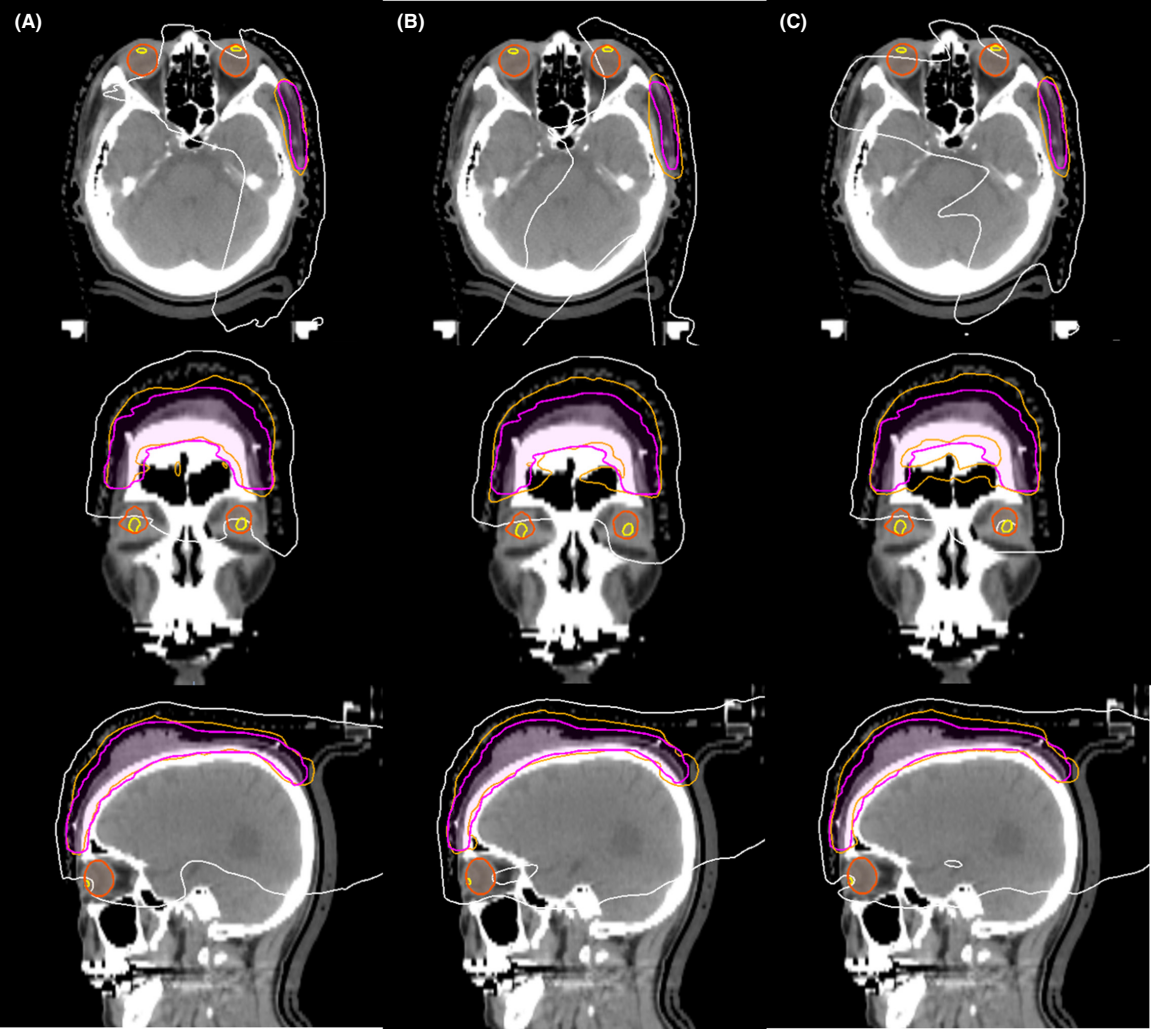
Dose distributions of the three plans in patient 4. (A) Volumetric modulated arc therapy, (B) 5‐field intensity modulated radiation therapy (IMRT) and (C) 9‐field IMRT. The planning target volume is rendered in pink. Isodose lines of 10 Gy and 95% of the prescribed dose are rendered in white and orange respectively.

The mean dosimetric values and dose–volume histograms for the three treatment plans in our four patients are shown in Table 3 and Figure 2 respectively. All PTV dose constraints were satisfied in all plans, except for *V*_110%_ in the 5‐field IMRT plans for patients 1 and 2. The PTV dose parameters of the VMAT plan were similar to those of the 9‐field IMRT plan. All OAR dose constraints were satisfied in both the VMAT and 9‐field IMRT plans, except for V_20 Gy_ in the brain in the 9‐field IMRT plan for patient 1. Regarding the 5‐field IMRT plans, the V_60 Gy_, V_45 Gy_ and V_20 Gy_ dose constraints and mean dose to the brain were not satisfied in patient 1, and the left lens dose constraints were not satisfied in patients 1 and 4. VMAT and 9‐field IMRT yielded similar mean dose parameters in the brain, and all mean dose parameters were equal to or lower than the corresponding parameters yielded by the 5‐field IMRT plan. Figure 3 shows the dose distributions of the three plans in patient 2. Compared with the other plans, 5‐field IMRT led to increased low‐dose spreading in the brain. Figure 4 shows dose distributions of the three plans in patient 4. VMAT and 9‐field IMRT plans effectively reduced the dose to the left lens in the patient with a PTV located near the left lens.

**Table 3:** Dosimetric results for three treatment plans of VMAT, 5‐field and 9‐field IMRT

Structure	Dosimetry	Mean value (range)		
		VMAT	5‐Field IMRT	9‐Field IMRT
PTV	*V*_110%_ (%)	0.13 (0.00–0.41)	3.89 (0.01–5.76)	1.43 (0.05–4.69)
	*D*_95%_ (%)	95.00	95.00	95.00
	*V*_93%_ (%)	98.29 (98.18–98.40)	98.11 (97.94–98.24)	98.06 (97.83–98.30)
	HI	0.14 (0.11–0.15)	0.16 (0.13–0.18)	0.15 (0.14–0.17)
	CI_100%_	0.63 (0.57–0.69)	0.61 (0.55–0.66)	0.64 (0.60–0.70)
	CI_98%_	0.72 (0.68–0.75)	0.66 (0.59–0.71)	0.70 (0.63–0.73)
	CI_95%_	0.74 (0.68–0.79)	0.67 (0.60–0.73)	0.71 (0.64–0.76)
Brain	*V*_60 Gy_ (%)	0 (0.00–0.00)	0.02 (0.00–0.08)	0 (0.00–0.00)
	*V*_45 Gy_ (%)	6.22 (2.63–9.09)	15.73 (4.61–32.09)	7.22 (2.59–11.89)
	*V*_20 Gy_ (%)	40.26 (20.22–58.22)	51.94 (20.84–88.30)	41.06 (18.33–61.61)
	*D*_mean_ (Gy)	18.33 (10.49–23.62)	23.49 (11.10–36.47)	19.40 (10.55–27.13)
Spinal cord	*D*_max_ (Gy)	3.20 (0.33–10.42)	5.84 (0.56–18.62)	6.98 (0.66–23.26)
Brain stem	*D*_max_ (Gy)	8.12 (1.84–11.63)	15.86 (1.98–33.48)	10.17 (2.16–17.85)
Optic chiasm	*D*_max_ (Gy)	6.38 (1.29–11.93)	9.05 (2.05–12.60)	6.95 (2.13–10.31)
Eye (R)	*D*_max_ (Gy)	18.90 (1.37–35.84)	18.02 (1.45–38.91)	18.22 (1.45–35.82)
Eye (L)	*D*_max_ (Gy)	24.71 (1.35–40.60)	21.79 (1.52–36.83)	20.53 (1.38–35.22)
Lens (R)	*D*_max_ (Gy)	5.42 (0.67–9.37)	5.33 (0.87–9.53)	5.39 (0.88–9.04)
Lens (L)	*D*_max_ (Gy)	7.14 (0.79–9.75)	9.59 (0.87–15.37)	6.63 (0.97–9.85)
Optic nerve (R)	*D*_max_ (Gy)	8.06 (1.19–16.41)	7.27 (2.07–11.23)	9.23 (2.06–18.46)
Optic nerve (L)	*D*_max_ (Gy)	10.34 (1.22–17.48)	7.40 (2.12–11.42)	10.44 (2.07–20.55)

VMAT, volumetric modulated arc therapy; IMRT, intensity modulated radiation therapy; PTV, planning target volume; *V*_*x*%_, fractional volume receiving *x*% of the prescribed dose; *D*_*x*%_, absorbed dose received by *x*% of PTV; HI, homogeneity index; CI, conformity index; *V*_*x* Gy_, fractional volume receiving *x* Gy; *D*_mean_, mean dose; *D*_max_, maximum dose.

**Figure 5:**
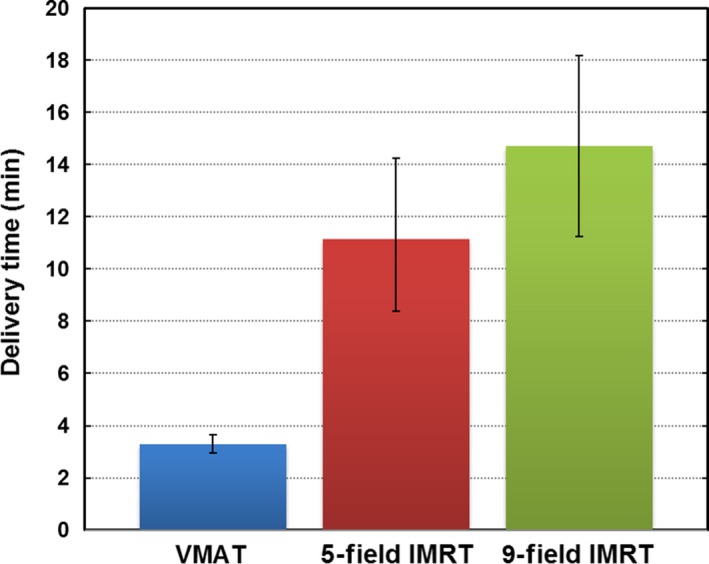
Delivery times of the VMAT, 5‐field and 9‐field IMRT plans in four patients. Delivery time was defined as the time between initiation of the first port and termination of the final port. Bars indicate the mean values and error bars represent data ranges. VMAT, volumetric modulated therapy; IMRT, intensity modulated radiation therapy.

The mean delivery times of the VMAT, 5‐field IMRT and 9‐field IMRT plans were 3.3, 11.1 and 14.7 min respectively (Fig. 5). The corresponding mean MUs were 1077, 823 and 935. Furthermore, the doses met the verification criteria.

## Discussion

Our study, which was based on the XVMC algorithm, revealed that VMAT provided comparable plan quality to 9‐field IMRT in terms of dose homogeneity and conformity of the PTV. These results were consistent with those of a previous study based on AAA by Hu et al.^8^ for diffuse sebaceous carcinoma. In their study, they employed non‐coplanar VMAT while dividing the target into several parts. We obtained acceptable dose homogeneity and conformity of PTV by using a simplified coplanar VMAT technique with a two‐arc approach, single isocentre and fixed collimator angle. This technical advantage may be due to the linear accelerator we used, as it was equipped with a state‐of‐the‐art MLC with lower leaf transmission, increased leaf speed and longer leaf travel than a standard MLC.^21^, ^22^ We performed dose calculation using dose to medium. As the difference between dose to medium and dose to water was approximately 5%,^23^ similar results were expected even if dose to water was used.

One of the biggest concerns during radiation therapy of scalp angiosarcoma is the dose to radiosensitive organs, namely the brain and lenses.^3^, ^5^ Although optimal dose constraints are under investigation, it is well known that maintaining minimal dose volumes to normal brain tissue is important to avoid neurocognitive dysfunction after radiation therapy.^24^ Similarly, the lens is one of the most radiosensitive tissues in the body; the threshold dose to the lens for cataract formation was reported to be 10 Gy.^25^ Similar to the dose distribution of the PTV, our study suggested that VMAT OAR doses were comparable to those of 9‐field IMRT. Furthermore, our study suggested that in certain patients, the use of VMAT or 9‐field IMRT might have reduced the radiation doses to the OARs, compared to 5‐field IMRT. As rotational irradiation techniques and a larger number of IMRT fields increase the number of beamlets being delivered tangentially to the scalp, VMAT and 9‐field IMRT may have an advantage over 5‐field IMRT in terms of dose distributions.^8^

One of the drawbacks of IMRT is a prolonged treatment time,^7^ which can be disadvantageous in certain aspects. At the institutional level, it may limit the number of patients who can be treated due to the increased machine occupancy time.^6^ From the patient's perspective, it may increase their discomfort and the risk of intra‐fraction movement of the target volumes and OARs.^26^, ^27^ A prolonged treatment time may lead to some distress among elderly patients, who comprise the majority of those affected by scalp angiosarcoma. Furthermore, it was suggested that prolonging the treatment time might result in the theoretical reduction in tumour control due to a higher number of opportunities for DNA repair and proliferation of the tumour.^6^ In our study, VMAT allowed a shorter delivery time, compared to IMRT, with a similar MU. The reason for the shorter delivery time with VMAT is that irradiation does not stop while the gantry is rotated,^6^ and introducing VMAT as a treatment for scalp angiosarcoma may resolve the disadvantages arising from prolonged IMRT sessions. The use of one‐arc VMAT may shorten the delivery time further; however, in our preliminary evaluation, we found that the dose distribution of one‐arc VMAT was inferior to that of two‐arc VMAT. This finding was consistent with that of Dai et al.^28^ who found the dosimetric benefit of two‐arc VMAT over one arc for the treatment of head and neck cancer. Therefore, we used two‐arc VMAT for this study.

One limitation of our study was the small number of patients. Although we compared plan quality with respect to PTV and OAR dose parameters, our sample size hindered our ability to perform a statistical evaluation of differences among the three irradiation techniques. Further investigations to address this issue are underway. Another limitation of our study was the potential for inter‐planner variability during dose optimisation. Although we defined various other specifications in this study, we did not establish a method for dose optimisation. Therefore, the results could have been influenced by individual planners’ preferences and/or arbitrariness. The use of an automated radiation therapy planning system is currently under investigation, and this issue should be addressed in the future.^29^

## Conclusions

Our data, which were obtained using a high‐accuracy dose calculation algorithm, XVMC, validate the use of VMAT over IMRT for the treatment of scalp angiosarcoma. Qualitatively, VMAT and 9‐field IMRT plans were similar in terms of doses to both the PTV and OARs. Additionally, VMAT was associated with a shorter delivery time, compared to IMRT. We therefore recommend VMAT as a sophisticated irradiation technique for the treatment of scalp angiosarcoma.

## Conflict of Interest

The authors report no conflicts of interest with respect to this research.
